# Quantitative nontumorous and tumorous human brain tissue assessment using microstructural co- and cross-polarized optical coherence tomography

**DOI:** 10.1038/s41598-019-38493-y

**Published:** 2019-02-14

**Authors:** Konstantin S. Yashin, Elena B. Kiseleva, Alexander A. Moiseev, Sergey S. Kuznetsov, Lidia B. Timofeeva, Nadezhda P. Pavlova, Grigory V. Gelikonov, Igor А. Medyanik, Leonid Ya. Kravets, Elena V. Zagaynova, Natalia D. Gladkova

**Affiliations:** 1 Privolzhsky Research Medical University, 603950 Minin and Pozharsky Sq., 10/1, Nizhny Novgorod, Russia; 2 Institute of Applied Physics, Russian Academy of Sciences, 603950 Ulyanova Str., 46, Nizhny Novgorod, Russia

## Abstract

Optical coherence tomography (OCT) is a promising method for detecting cancer margins during tumor resection. This study focused on differentiating tumorous from nontumorous tissues in human brain tissues using cross-polarization OCT (CP OCT). The study was performed on fresh *ex vivo* human brain tissues from 30 patients with high- and low-grade gliomas. Different tissue types that neurosurgeons should clearly distinguish during surgery, such as the cortex, white matter, necrosis and tumorous tissue, were separately analyzed. Based on volumetric CP OCT data, tumorous and normal brain tissue were differentiated using two optical coefficients — attenuation and forward cross-scattering. Compared with white matter, tumorous tissue without necrotic areas had significantly lower optical attenuation and forward cross-scattering values. The presence of particular morphological patterns, such as necrosis and injured myelinated fibers, can lead to dramatic changes in coefficient values and create some difficulties in differentiating between tissues. Color-coded CP OCT maps based on optical coefficients provided a visual assessment of the tissue. This study demonstrated the high translational potential of CP OCT in differentiating tumorous tissue from white matter. The clinical use of CP OCT during surgery in patients with gliomas could increase the extent of tumor resection and improve overall and progression-free survival.

## Introduction

In the adult population, gliomas are the most common central nervous system tumors (34%)^1,2^. The goal of modern glioma surgery is achieving maximum resection while preserving eloquent brain areas^3^. The extent of tumor resection is associated with improved overall and progression-free survival, most significantly for low-grade gliomas^3–10^. Due to infiltrative growth into surrounding brain tissue, differentiating tumorous and nontumorous tissue to achieve total tumor resection is difficult^6,7,11^. Intraoperative imaging technologies such as 5-ALA-guided resection and intraoperative MRI may be beneficial in maximizing the extent of resection^12–15^, but these methods also have some limitations (for example, the necessity of using contrast agents). Moreover, despite a substantial number of studies, the effectiveness of these technologies in maximizing the extent of glioma resection is based on low to very low quality evidence^16^.

Progress in optical bioimaging techniques opens the door to new opportunities in neurological surgical guidance during brain tumor removal^17,18^. One of the most promising methods is optical coherence tomography (OCT)^18,19^, a medical imaging technique for obtaining microscopic images of biological tissue in different medical disciplines, including ophthalmology, endovascular surgery, dermatology, and gastroenterology.

OCT is based on low-coherence interferometry in the near IR range of wavelengths (700–1,300 nm) to obtain images of tissue microstructure in real time with micron resolution at depths of 1–2 mm^20^. Although the resolution of OCT is insufficient for the identification of single tumor cells within peritumoral brain tissue at the edges of the tumor cavity^21^, OCT has a great ability to detect myelinated axons, thereby delineating glioma margins (for low- and high-grade gliomas) from white matter^22,23^. Moreover, OCT can image at a distance, allowing the integration of OCT into the optical path of surgical microscopes^24,25^. Additionally, intraoperative identification of tumor margins during glioma surgery is possible by using a neuroendoscopic probe^23,26^.

Tumorous and nontumorous tissues can be differentiated by OCT using qualitative^23,27^ or quantitative assessment^22,23,28,29^ of OCT images. Quantitative assessment is based on the calculation of different optical coefficients and is considered more objective. Quantitative assessment also allows the construction of color-coded maps of calculated coefficients^23,28^, making tumorous and nontumorous regions more obvious by their visual distinguishing characteristics than the conventional color scheme of OCT. Despite significant progress in OCT image acquisition and processing, the criteria for differentiation between tissue types are not clear-cut. In addition, the tissue types that should be identified during tumor resection are not limited by the terms “tumorous” and “nontumorous” (most often imply only white matter) tissues and comprise gray matter (both cortical and subcortical), white matter, tumor tissue (grade I-IV), and necrosis (spontaneous tumor necrosis, radiation and coagulative necrosis). Therefore, the potential and limitations of the tissue differentiation method remain unclear.

Conventional intensity-based OCT has been used in the visualization of stratified tissue types, such as the retina. However, OCT can be less capable of detecting pathological changes in structureless tissues, such as the brain, due to a lack of tissue-specific contrast. However, the potential of OCT has been constantly increasing through the development of functional extensions of OCT (e.g., Doppler/angiographic OCT and polarization-sensitive (PS) OCT). PS OCT can detect polarization state changes in the probe light in tissue, thereby generating tissue-specific contrast and extending quantitative measurements of OCT signal^30,31^. PS OCT is based on the birefringence of the medium (mainly associated with interaction of light and anisotropic tissue structures) and provides better visualization of elongated structures that have longitudinal dimensions much larger their transverse dimensions, such as myelinated nerve fibers^32,33^. Cross-polarization OCT (CP OCT) is a variant of PS OCT that allows imaging of initial polarization state changes due to both birefringence and cross-scattering in biological tissue^34,35^. In a previous study, tumorous tissue and white matter could be perfectly differentiated by visual assessment of CP OCT images^29^. The present work presents the potential of CP OCT to detect different tissue types by optical coefficient calculation (attenuation (ν) and forward cross-scattering (C)). Based on these coefficients, we created *en face* color-coded maps of tissues.

## Results

### CP OCT optical properties of different tissue types

#### White matter CP OCT features

White matter can be characterized by high attenuation (ν_WM_ = 8.5 mm^−1^) and forward cross-scattering (C_WM_ = 0.56 mm^−1^) properties. White matter and tumor tissue exhibit optical differences (Table 1). Both attenuation and forward cross-scattering coefficients showed significant differences between white matter (with maximal coefficient values) and astrocytomas grade I–IV (p < 0.001) regardless of whether a necrotic component was present. In addition, the C_WM_ value was substantially greater than C_GrI-III_ and C_GrIV_ when the relevant attenuation coefficient values (C_WM_/C_GrI-III_(C_GrIV_) ≈ 10 versus ν_WM/_ν_GrI-III_(ν_GrIV_) > 1) were compared.

**Table 1:** Optical coefficients difference between tumorous and non-tumorous tissue types.

		Coefficient Me [Q_1_;Q_3_]^a^	p (versus white matter)^b^	p (versus cortex)^b^
	attenuation (ν), mm ^−1^			
Normal tissue	White matter (n = 16)	8.5 [8.2; 9.3]	—	<*0*.*0001*
	Cortex (n = 16)	5.0 [3.2; 5.5]	<*0*.*0001*	—
Tumor subtype 1 (T1) without necrotic areas	Astrocytoma Grade I–III (n = 28)	3.0 [2.6; 3.5]	<*0*.*0001*	<*0*.*0001*
	Glioblastoma Grade IV without necrotic areas (n = 16)	3.15 [2.6; 4.2]	<*0*.*0001*	*0*.*015*
Tumor subtype 2 (T2) with necrotic areas	Glioblastoma Grade IV with necrotic areas (n = 28)	6.3 [5.4; 6.8]	<*0*.*0001*	<*0*.*001*
	Necrosis (n = 19)	7.5 [5.3;7.7]	<*0*.*001*	<*0*.*0001*
	forward cross-scattering (C), mm ^−1^			
Normal tissue	White matter (n = 16)	0.56 [0.31; 0.89]	—	<*0*.*0001*
	Cortex (n = 16)	0.022 [0.015; 0.043]	<*0*.*0001*	—
Tumor subtype 1 (T1) without necrotic areas	Astrocytoma Grade I–III (n = 28)	0.017 [0.014; 0.019]	<*0*.*0001*	0.022
	Glioblastoma Grade IV without necrotic areas (n = 16)	0.019 [0.015; 0.024]	<*0*.*0001*	0.44
Tumor subtype 2 (T2) with necrotic areas	Glioblastoma Grade IV with necrotic areas (n = 28)	0.13 [0.08; 0.19]	<*0*.*0001*	<*0*.*0001*
	Necrosis (n = 19)	0.18 [0.11; 0.32]	<*0*.*0001*	<*0*.*0001*

^a^Me [Q_1_;Q_3_]: Me – median; [Q1;Q3] – 25th and 75th percentiles values respectively.

^b^U-test Mann-Whitney.

#### Cortex CP OCT features

**Figure 1:**
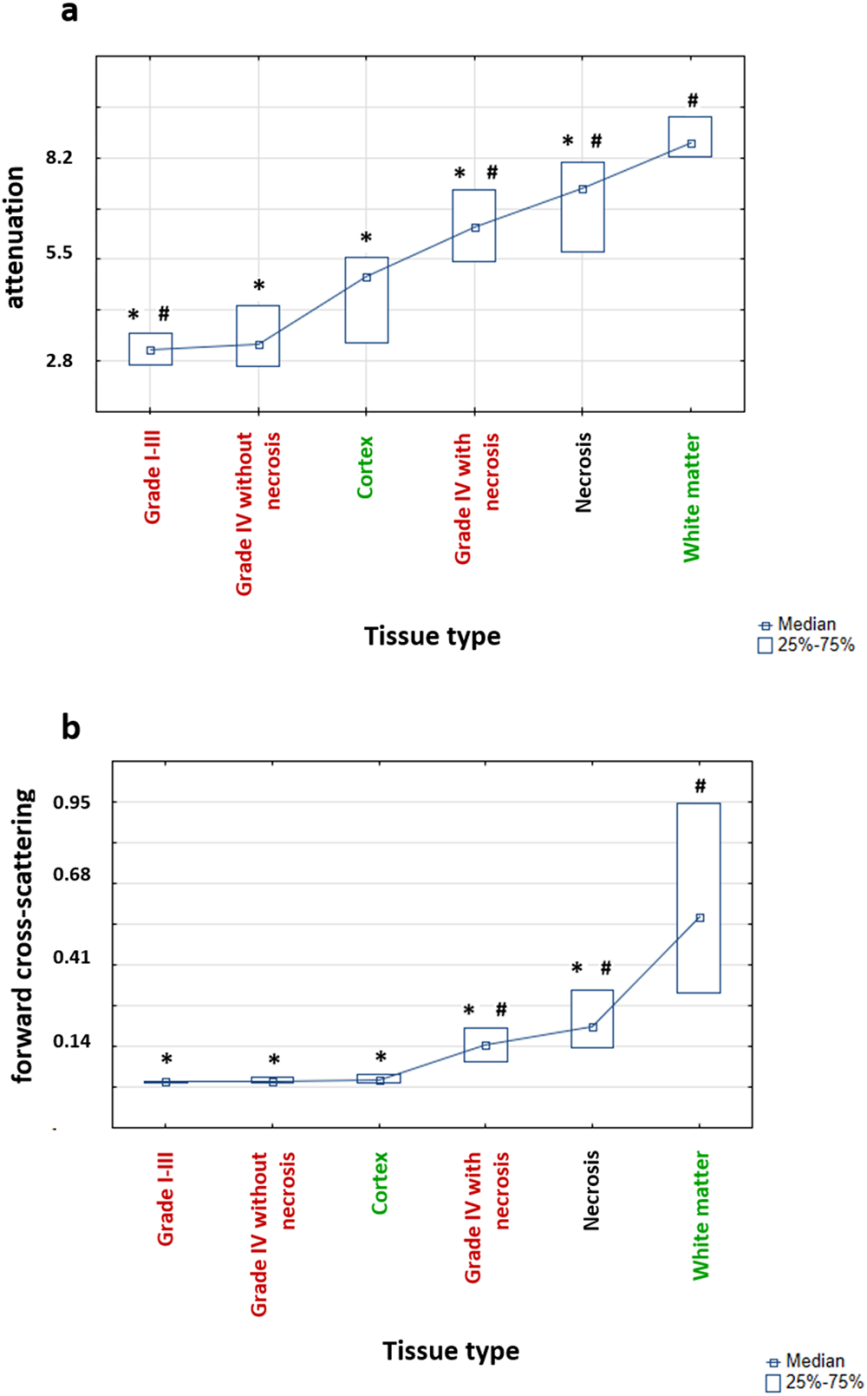
The distribution of attenuation (**a**) and forward cross-scattering (**b**) coefficients values between different tissue types. Data are presented as medians with 25th and 75th percentile values. ^*^Significant differences between white matter and other groups; ^#^significant differences between the cortex and other groups.

The cortex was well differentiated from white matter using both attenuation and forward cross-scattering coefficients (ν_WM_ = 8.5 mm^−1^ and C_WM_ = 0.56 mm^−1^ versus ν_CTX_ = 2.5 mm^−1^ and C_CTX_ = 0.022 mm^−1^, p < 0.0001). Compared with white matter, cortex and tumor tissue exhibited no clear optical differences (Table 1). Although the attenuation coefficient of the cortex was significantly higher than that of tumor tissue without necrotic areas and lower than that of tissue with necrosis, there were no clear differences in the forward cross-scattering coefficient between the cortex and tumor except for tumor tissues with necrotic components. Figure 1 shows that in both cases (attenuation and cross-scattering), the cortex coefficient values are placed between tumor tissue without necrotic components and necrosis.

#### Tumor CP OCT features

Depending on the presence of necrotic areas, two subtypes of tumor tissue with different optical properties could be separated as follows: (1) glioma tissue without necrotic areas (“grade I-III” and “glioblastoma grade IV without necrotic areas”) — ν_T1_ = 3.0 [2.6; 3.56] mm^−1^ and C_T1_ = 0.017 [0.014; 0.019] mm^−1^; (2) tissue with partial or total necrosis (“glioblastoma grade IV with necrotic areas” and “necrosis”) — ν_T1_ = 5.5 [5.3; 7.67] mm^−1^ and C_T1_ = 0.18 [0.11; 0.32] mm^−1^. Attenuation and scattering in CP were significantly higher (p < 0.0001) for tumor tissue with necrotic areas than for glioma without necrosis. This difference was more obvious in CP. Glioma tissue with necrosis exhibited optical properties similar to those of white matter (particularly attenuation), whereas glioma tissue without necrosis exhibited properties similar to those of the cortex.

### Optical coefficient thresholds for distinguishing white matter and tumor tissue

**Figure 2:**
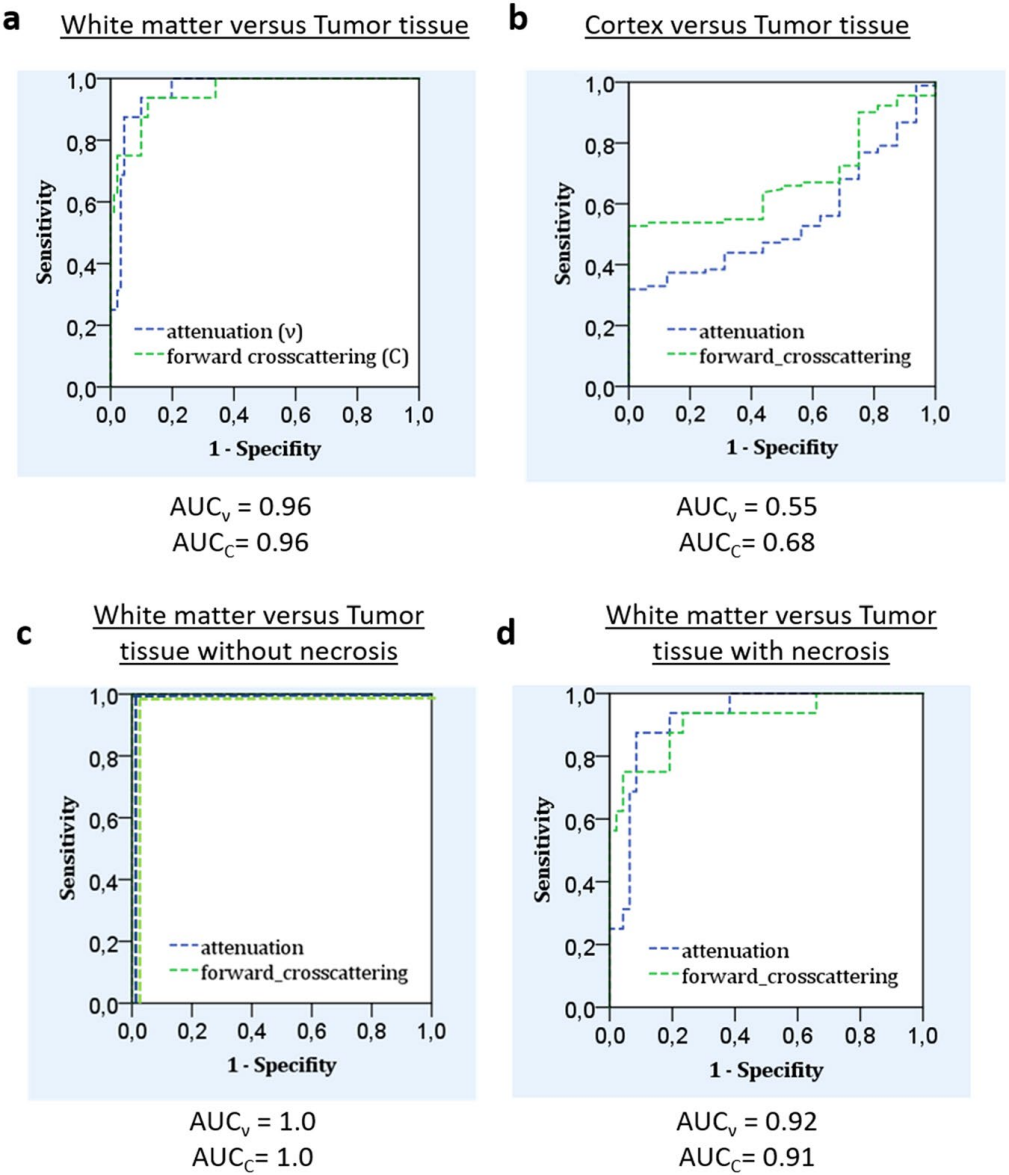
ROC analysis of optical coefficient data for white matter and tumors in general (**a**), cortex and tumor (**b**), white matter and tumor without a necrosis component (**c**), white matter and tumor with a necrosis component (**d**).

ROC analysis revealed that both coefficients have good diagnostic value in differentiating between tumor tissue and white matter (Fig. 2a) in contrast to the cortex (Fig. 1b). Therefore, distinguishing between tumor tissue and white matter was less easy in the presence of necrotic areas in tumor tissue (Fig. 2d). All tissues in the data set were used to determine the optimal threshold value with maximum sensitivity and specificity for both attenuation and forward cross-scattering coefficients. In general, with optimal optical coefficient thresholds of ν = 8.2 mm^−1^ and C = 0.026 mm^−1^, the specificity was 81.3% and 87.5%, and the sensitivity was 95.6% and 90.1%, respectively. For glioma tissue without necrosis, the specificity and sensitivity were 100% (Table 2).

**Table 2:** Sensitivity, specificity and diagnostic accuracy of the test “white matter versus tumor” according to thresholds of attenuation and forward cross-scattering coefficients.

	Coefficient threshold value, mm^−1^	Sensitivity. %	Specificity. %	Diagnostic accuracy. %
attenuation				
All tumors	8.2	95.6	81.3	93.5
Tumor without necrosis (T1)	6.6	100	100	100
Tumor with necrosis (T2)	8.2	91.5	81	89
forward cross-scattering				
All tumors	0.26	90.1	87.5	90
Tumor without necrosis (T1)	0.09	100	100	100
Tumor with necrosis (T2)	0.26	81	87.5	82.5

### Optical coefficients color-coded maps of tumorous and nontumorous tissues and their margins

**Figure 3:**
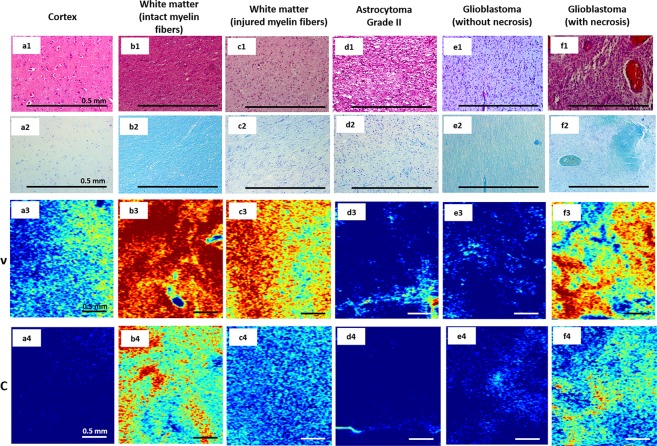
Color-coded maps and corresponding histology of tumorous and normal brain tissues. Based on attenuation (ν) (**a3**–**f3**) and forward cross-scattering (С) (**a4**–**f4**) coefficients, en face color-coded maps of the cortex (**a1**–**a4**), white matter with intact (**b1**–**b4**) and injured (**c1**–**c4**) myelin fibers, astrocytoma grade II (**d1**–**d4**), glioblastoma grade IV with necrosis (**e1**–**e4**) and without necrosis (**f1**–**f4**) corresponding to histology in HE (**a1**–**f1**) and Luxol blue (**a2**–**f2**) staining.

Compared with intensive *en face* CP OCT images, color-coded maps of glioma and white matter based on different optical coefficients (Fig. 3a3–f3, a4–f4) were more representative for visual assessment of the tissue. The color set of each map reflected the values of the corresponding optical coefficient; therefore, the areas of high attenuation or forward cross-scattering (myelin fibers, necrosis) appeared in light tones, and the areas of low attenuation or forward cross-scattering (cell clusters) appeared in darker tones.

**Figure 4:**
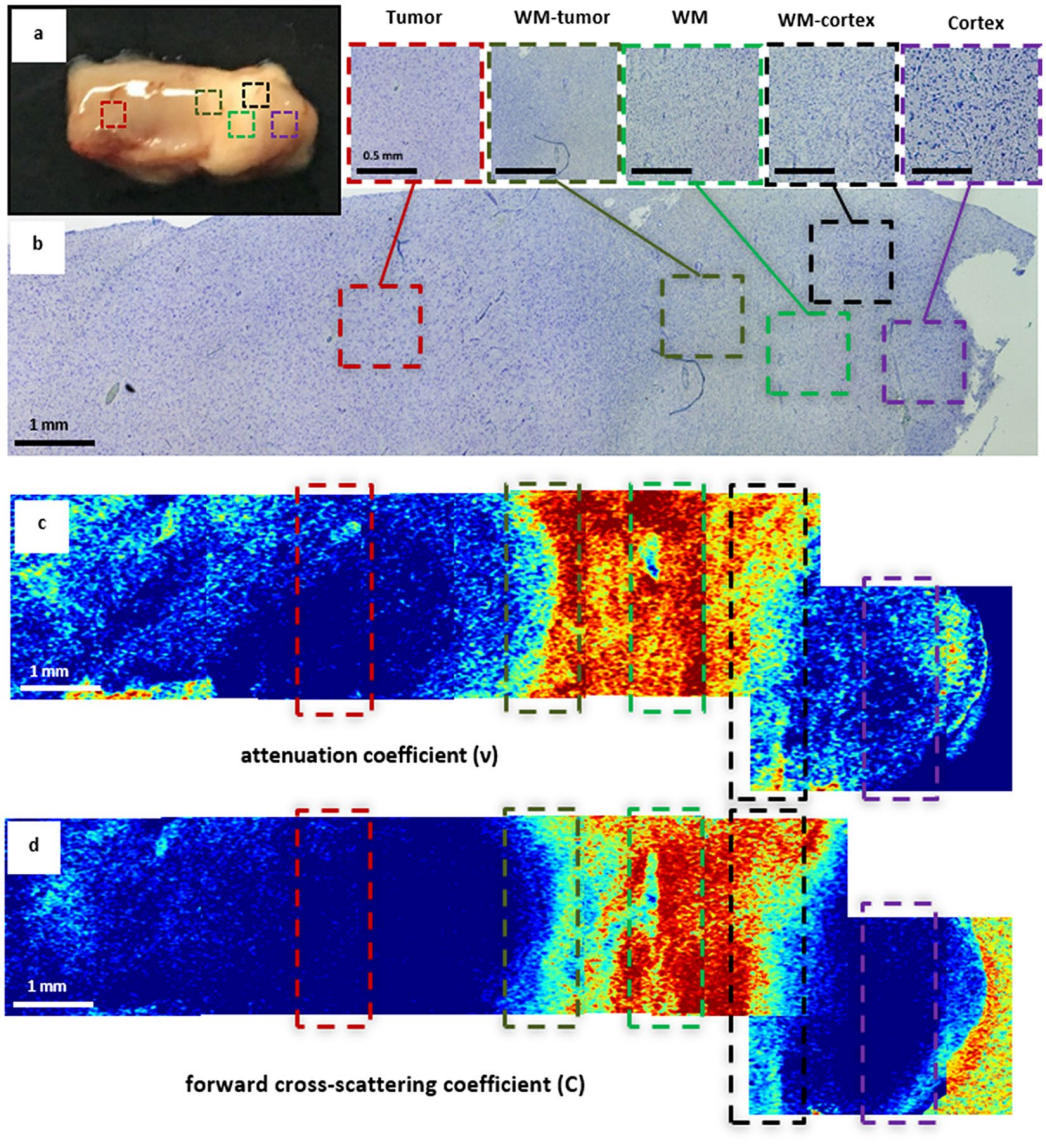
Color-coded maps based on attenuation coefficients (**c**) and forward cross-scattering coefficients on a logarithmic scale (**d**) with corresponding histology (**b**) of a brain specimen (**a**). The areas of interest are marked on each image by different colors: white matter (green dotted line), astrocytoma Grade II (red dotted line), cortex (violet dotted line), margins between white matter and tumors (dark green dotted line); cortex and tumors (black dotted line), and corresponding histology (**b**) where the areas of interest are presented under high magnification and marked with the corresponding colors. Histological images (**b**), Luxol blue staining. WM — white matter.

Thus, on the attenuation map, white matter formed by densely packed myelin fibers appeared red and yellow (Fig. 3b4), and tumors formed by cell clusters appeared dark or pale blue (Fig. 3d3,e3). On the forward cross-scattering map, white matter appeared pale blue to red (Fig. 3b4), whereas tumors appeared dark or pale blue (Fig. 3d4,e4). These differences are more evident on color-coded maps of the margin between tumors and white matter. The dividing line was more clearly visualized by every coefficient (Fig. 4c,d, dark green dotted line). Nevertheless, on two separate occasions, the white matter and tumor tissue looked similar on OCT images as follows: (1) white matter in the peritumoral area, particularly in high-grade gliomas, could be characterized by a decreased OCT signal because of myelin fiber destruction and/or intense edema (Fig. 3c3,c4); in this area, tumor cells could likely persist; and (2) tumor tissue with necrotic areas (e.g., glioblastoma core, tumor tissue after partial bipolar coagulation) had high attenuation and forward cross-scattering (Fig. 3f3,f4).

The cortex comprises neurons and glial cells surrounded by uncompacted myelin fibers. For this reason, white matter on attenuation maps was represented by the whole color set, but on forward cross-scattering, it was mainly blue, similar to astrocytomas (Fig. 3a3,a4). Thus, the identification of a well-marked margin could be difficult. Additionally, the cortex could be differentiated from white matter by the suggested coefficients. Visually, the cortex was well defined on color-coded maps that were generated across the gray matter–white matter margin (Fig. 4c,d, black dotted line). On the color-coded maps, the margin between the tumor and white matter was clearly visualized by every coefficient (Fig. 4c–e, dark green dotted line).

## Discussion

Active application of OCT to clinical practice is determined by the clear benefits of this method, such as using near infrared light sources with no risk of tissue damage, high resolution (~10 micron), no need for contrast agents, imaging depths of more than 1 mm, and imaging at a distance, therefore enabling integration into a surgical microscope or endoscope^17^. The further development of OCT can be realized by adding new technical advances (functional extensions) and enhancing OCT signal interpretation.

Initial studies by various research groups aimed to determine qualitative (visual) criteria for the differential diagnosis of tumors and normal brain tissue according to the OCT signal characteristics of intensity images (for CP OCT, intensity imaging corresponds to imaging in copolarization)^22,27,36^. Based on the surgeon’s opinion, this approach seems to be subjective, and unfortunately, clinically approved OCT systems can provide only visual assessment of OCT signals^23–26^.

Great advances in the adaptation of OCT into clinical practice are connected to a more objective quantitative evaluation of the OCT signal with a determination of various optical coefficients, such as backscattering^37^ and attenuation^22,23,28^ coefficients. Kut *et al*.^23^ obtained the following attenuation coefficient values relevant to our data: for normal white matter — 6.2 ± 0.8 mm^−1^; for high malignancy gliomas — 3.9 ± 1.6 mm^−1^; in the infiltration zone — 7.1 ± 1.0 mm^−1^; for low malignancy gliomas — 4.0 ± 1.4 mm^−1^; and in the infiltration zone — 2.7 ± 1.0 mm^−1^. Kut *et al*. demonstrated high diagnostic accuracy of OCT for tumors against white matter based on the optical attenuation threshold of 5.5 mm^−1^. For high-grade patients, the sensitivity and specificity were 92% and 100%, respectively; for low-grade patients, the sensitivity and specificity were 100% and 80%, respectively.

In our study, attenuation coefficient for brain tissues had different absolute values (see Table 1) in comparison with Kut *et al*.^23^ results. The differences in the obtained data in our study could be explained by the individual specifications of OCT devices and different approaches to signal processing. In our study, the diagnostic threshold of attenuation coefficient was 8.2 mm^−1^ for patients of all grades (sensitivity/specificity: 95.6%/81.3%, respectively) and 6.1 mm^−1^ for low-grade patients (sensitivity/specificity: 100%/100%, respectively).

The advantage of using cross-scattering coefficient in our study consisted in acquiring a significant difference in its absolute value in the differentiated tissues and its higher specificity in comparison with attenuation coefficient in the diagnosis of gliomas. The difference in the absolute values of the cross-scattering coefficient in the white matter and gliomas was tenfold (0.56 mm^−1^ vs 0.06 mm^−1^, respectively), while the difference in the attenuation coefficient was less than twofold (8.5 mm^−1^ vs 5.1 mm^−1^, respectively). The diagnostic threshold of cross-scattering coefficient was 0.26 mm^−1^ for patients of all grades (sensitivity/specificity: 90.1%/87.5%, respectively) and 0.09 mm^−1^ for low-grade patients (sensitivity/specificity: 100%/100%, respectively). Сross-scattering coefficient demonstrated higher specificity but lower sensitivity in patients of all grades with gliomas in comparison with attenuation coefficient. Further studies should focus on identifying additional optical coefficients and increasing diagnostic accuracy by using coefficient combinations.

These promising results can be explained by the tissue structure organization. Tumorous tissue can be represented as a massive assembly of tumor cells, which are characterized by low attenuation and forward cross-scattering, regardless of cell density, in contrast to the high attenuation and forward cross-scattering of myelin. Despite the high diagnostic accuracy of OCT in differentiating between white matter and tumors, difficulties can occur in distinguishing between white matter (intact or with structureless myelin) and tumorous tissue with necrosis due to their similar optical coefficient values and color-coded map overviews (Fig. 3b3 vs f3, b4 vs f4). Therefore, this issue necessitates the division of white matter and tumors into several types as follows: (1) tumors according to the presence of necrotic areas and (2) white matter according to myelin fiber organization.

Rather than dividing tumorous tissue specimens by low-grade and high-grade gliomas (as generally accepted), dividing all specimens by the presence/absence of necrotic areas is necessary due to the substantial optical differences between these tissues (in the same way as histological differences). Tumorous tissue without necrosis forms the typical structure of astrocytomas grade I-III and can also be found in the infiltration zone of glioblastoma. Necrotic areas that can dramatically increase attenuation (forward cross-scattering) are generally present (1) in the glioblastoma core (central part of the tumor), (2) in tissue after radiotherapy (e.g., in recurrent astrocytoma after combination treatment) and (3) after bipolar coagulation during tumor resection (total necrosis).

The high attenuation (forward cross-scattering) of white matter is due to high-density packaging of myelin fibers. Some events, such as structural damage to myelin or cerebral edema related to tumor growth, can lead to a significant decrease in attenuation and forward cross-scattering. These myelin changes are typical in the peritumoral area of high-grade gliomas (especially glioblastoma). Notably, “partisan” tumor cells but not massive tumor cell clusters are likely present in this area.

In all doubtful cases, the surgeon could primarily draw on the patient’s clinical data, localization of the OCT scanning area and distance to eloquent brain areas, which are responsible for critical brain functions such as movement and speech. For example, if preoperative clinical data indicate low-grade glioma, no necrotic areas or myelin damage are expected, and the surgeon should not suspect the presence of necrotic areas or dramatic changes in myelin structure in the peritumoral area. In glioblastoma, the white matter in the peritumoral area could be characterized by low attenuation and forward cross-scattering; thus, the decision regarding the extent of resection in this area should be primarily based on the distance to eloquent brain areas. Additionally, using different optical thresholds is a possibility as the surgical situation demands, e.g., choosing a threshold of 6.1 mm^−1^ in astrocytoma grade I-III and 8.2 mm^−1^ in glioblastoma or recurrent astrocytoma after radiotherapy.

Hemorrhage can compromise the interpretability of OCT signals^22^; therefore, before scanning, hemorrhage and necrotic areas associated with bipolar coagulation should be removed. For intraoperative surgical guidelines, the revealed optical thresholds of each coefficient should be corrected because there are differences between *ex vivo* and *in vivo* coefficients^38^.

In contrast to white matter, the cortex does not contain densely packed myelin fibers that can lead to difficulties in distinguishing between the cortex and tumor tissue using optical coefficients, even though there are significant differences between these tissues. On *in vivo* cross-sectional OCT scans, the cortex is characterized by a specific vertical striation arising from the “shadows” of blood vessels located just under the tissue surface. Visual assessment of OCT signals during *in vivo* scanning essentially aids in distinguishing the tumor from the cortex by losing “normal” attenuation^22,38^. Additional *in vivo* studies could allow us to more clearly determine optical differences between the cortex and tumor and define quantitative OCT criteria as well as correct optical thresholds for distinguishing white matter and tumors^38^.

Color-coded maps are based on the combination of qualitative and quantitative assessment of OCT; therefore, compared to *en face* OCT images, these maps appear more representative in margin detection. Kut *et al*. presented color-coded maps using 3 colors; these authors used green, red, and yellow to denote white matter, glioma tissue, and the infiltrative zone, respectively^23^. In our study, the range of colors corresponding to optical coefficients was wider because optical thresholds were not used. Nevertheless, substantial evidence supports visually distinguishing between white matter and tumors or the cortex (gray matter). Although both coefficients have demonstrated clear borderlines, color-coded maps in CP appear more user friendly for practical use because they can more clearly represent the keystone structure — myelin and its condition. Currently, using color-coded maps for distinguishing between tumors, cortex, necrosis and white matter with injured myelin fibers is challenging.

The presented *ex vivo* CP OCT structural imaging and quantification method has some methodological limitations. For instance, a tissue’s optical properties may not be consistent in different situations from *in vivo* to *ex vivo*, and consistent tissue properties cannot be assumed during surgical procedures. However, based on preliminary data from animal experiments^29^, general distinguishing features of different brain tissue types, such as signal intensity and homogeneity/heterogeneity, will be translated from *ex vivo* to *in vivo* studies. Additional correction of optical coefficient values obtained from *ex vivo* specimens will be performed during further intraoperative studies before being extrapolated to routine clinical practice.

Furthermore, the “normal” cortex and white matter used in the present study were from the area surrounding the tumor, which is often infiltrated with tumor cells and may have affected our results. In this study, we used hematoxylin and eosin (HE) staining, which does not provide complete information about the presence of tumor cells in the surrounding brain tissue. Immunohistochemistry for the common IDH (isocitrate dehydrogenase) mutation in IDH mutated tumors and MIB-1 (monoclonal antibody to Ki67) staining in IDH wild-type tumors would be more sensitive to low levels of tumor infiltration. Using these staining techniques in future studies will allow us to update the threshold for OCT coefficients to detect the infiltration zone.

Notably, in astrocytoma, the evaluation of all tumor cells is not possible due to its infiltrative and widespread growth (tumor cells can be discovered far away from the initial tumor and even in other hemispheres). Therefore, in glioma surgery, the term “maximal tumor resection” is controversial. In glioblastoma, neurosurgeons seek to resect the contrast-enhanced area shown on preoperative MRI. The remaining tumor could be targeted by radiation, chemotherapy or adjuvant therapy and other novel treatment modalities.

Nevertheless, OCT does not provide molecular information and cannot detect single tumor cells; it appears promising for navigation during neurosurgical procedures, such as glioma resection, stereotactic biopsy and electrode placement for deep brain stimulation. For clinical translation, an OCT imaging device can be integrated with standard operating theater equipment, such as surgical microscopes^39,40^ or needles for stereotactic procedures^41^. For the purposes of glioma surgery, CP mode is a good example of OCT functional extension, which can improve OCT data processing. Another promising OCT extension enables the detection of blood vessels and could prevent damage to these structures during stereotactic procedures.

Currently, there are two main intraoperative technologies, 5-ALA fluorescence and intraoperative MRI, that significantly enhance the identification of remaining tumor tissue and increase the survival rate. However, these methods have some limitations (e.g. the need for contrast agents and lack sufficient resolution), which necessitate the development of innovative technologies, such as confocal microscopy, multiphoton tomography, high-resolution OCT (capable of visualizing cells) or Raman spectroscopy, that are highly sensitive to different aspects of molecular and atomic structures. Unfortunately, all microscopic methods have a small scanning area (narrow field); therefore, scanning the whole brain tumor resection cavity will take a great deal of time. Additionally, every technology has its own limitations; for instance, OCT has insufficient resolution, and Raman spectroscopy has an intrinsically low signal. The development of multimodal systems can potentially overcome these limitations and provide fast brain tumor demarcation with high sensitivity and specificity.

In conclusion, OCT can be treated as a promising technique for efficiently distinguishing tumorous tissue from white matter during surgical resection of malignant brain tumors by using several optical coefficients and color-coded maps based on these tissues. This study has demonstrated the high translational potential of CP OCT that can perfectly represent the keystone structure of brain parenchyma — myelin and its condition. Unfortunately, OCT currently has difficulties in clearly distinguishing between the cortex and tumorous tissue. Therefore, further studies and technological refinement are needed to increase the diagnostic capabilities of OCT and to provide rapid and efficient detection of brain tumors during surgery.

## Materials and Methods

### Patients

*Ex vivo* specimens of different tissue types were obtained during tumor resection from 30 patients with gliomas of different degrees of malignancy as follows: astrocytoma grade I-II (n = 8), astrocytoma grade III (n = 7), and glioblastoma grade IV (n = 15).

**Figure 5:**
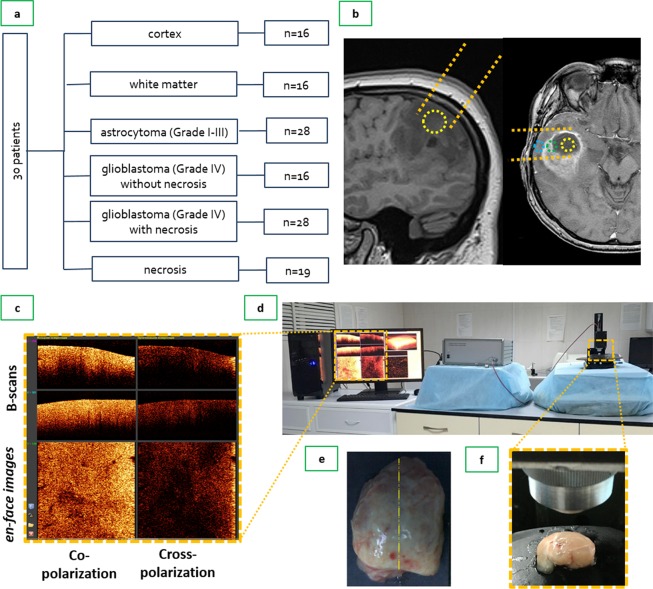
Design of the CP OCT study: (**а**) the study was performed on material from operative biopsies as follows: 30 patients with gliomas of different grades of malignancy; in total, 123 *ex vivo* specimens were analyzed; (**b**) MRI image of astrocytoma grade II (left) and glioblastoma grade IV (right) with outlining trajectory of surgical approach (orange dotted lines) and the areas of tissue sampling: area within the yellow dotted line — center of the tumor; area within green dotted line — peritumoral area; area within blue dotted line — edge of the resection (nontumorous tissue at the place of access to the tumor); (**c**) enlarged part of monitor with B-scans and *en face* OCT images in co- and cross-polarization; (**d**) working area for CP OCT scanning with the CP OCT device and on-mount optical probe; (**e**) resected specimen with schematic markings of the scanning area along the central line (yellow dotted line); (**f**) position of contactless forward-looking optic fiber CP OCT probe above the sample.

A surgical approach to tumors was performed using a frameless navigation system with uploaded functional MRI data and intraoperative neurophysiological monitoring (as well as “awake” surgery) for the preservation of motor and speech eloquent brain areas and white matter tracts. Along the trajectory of the surgical approach, nontumorous tissue in the peritumoral area that was routinely subjected to coagulation was accurately marked and removed (Fig. 5b). During resection, tumorous tissue specimens were also taken from different parts of the tumor.

All specimens were immediately placed in Petri dishes, covered with cotton moistened with cold saline solution and closed to prevent dehydration during delivery to the location of the OCT study, which took several minutes. Before OCT imaging, the tissue surface was cut to create a flat fresh surface on the sample; next, the sample was placed on a special motorized table for convenient movement under the OCT probe. The duration of the CP OCT study of one sample was approximately 10–15 min (including tissue preparation). The cerebral cortex is more sensitive to external conditions than white matter; therefore, we studied samples with gray matter first. Other specimens were left in a closed Petri dish.

No deterioration of patient condition was noted after surgery compared to that before surgery. The study was approved by the Ethical Committee of the Privolzhskiy Federal Research Medical Centre of the Ministry of Health of the Russian Federation, and informed consent was obtained from all patients. All methods were performed in accordance with the relevant guidelines and regulations.

### Cross-polarization OCT

The study was performed with a spectral-domain OCT device with the CP detection method developed in the Institute of Applied Physics of the Russian Academy of Sciences (Nizhny Novgorod, Russia)^42,43^. The utilized device has a common-path interferometric layout that operates at a 1.3 µm central wavelength with axial and lateral resolutions of 10 µm and 15 µm in air, respectively. The circular probing beam polarization state was maintained at the output of the flexible probe. The circular polarization state of the probing beam avoids dependence on probe-tissue mutual orientation. CP characterizes polarization state changes due to propagation through anisotropic media. The active polarization control system is based on analysis of the polarization state of light returned from the probe fiber tip. The system is based on the formation of the probe equivalent to a quarter-wave retarder plate with a 45° orientation of its optical axes with respect to the linearly polarized reference wave. This method was described in detail in a previous study^44^. Due to the use of active polarization control, the system is robust and does not require day-to-day maintenance. The device has a 20,000 А-scan/s scanning rate and performs 2D lateral scanning with a range of 2.4 × 2.4 mm^2^ to obtain a 3D distribution of backscattered light in the polarization parallel and orthogonal to the polarization of the probing beam. Scanning was performed in contactless mode (Fig. 5e). In total, 123 specimens were scanned and analyzed (Fig. 5а).

### Histological study

After imaging, the scanning area on the specimen was marked with histological ink; then, the specimen was fixed in 10% formalin for 48 hours and resectioned through the marked area such that the plane of histological sections corresponded to *en face* CP OCT images. A series of sections were stained with HE and observed by a pathologist. According to histological examination, all tissue samples were divided into 6 categories (Fig. 5a). In addition, Luxol blue staining was used to evaluate the presence of myelinated fibers in the samples. Histological slides were viewed and photographed with a microscope equipped with a digital camera (Leica DM 2500, DFC 245 C) in transmitted light. Two histopathologists independently evaluated pathological slides stained with HE and Luxol blue. The diagnosis coincided in 98% of cases.

### Optical coefficients

Polarization effects in the tissue may lead to the different attenuation of the signal in two orthogonal channels. This difference can be used as an additional characteristic of the tissue. It was expected that scattering and absorption on elongated features in the tissue should be equal for both utilized clockwise and counterclockwise circular polarizations. The assumed mechanism leading to the observed difference in signal attenuation was forward scattering with a change in the initial polarization state. For optically homogeneous tissue, such as brain, the attenuation coefficient can be estimated from the logarithm of the measured signal:

$$\mathrm{ln}({{\rm{I}}}_{{\rm{co}}}) \sim -\,2z{\rm{\nu }},$$

$$\mathrm{ln}({{\rm{I}}}_{{\rm{cross}}}) \sim -\,2{\rm{z}}({\rm{\nu }}-{\rm{C}}),$$

where I_co_ and I_cross_ are signals in co- and CP channels, respectively; ν and (ν-C) are attenuation coefficients in corresponding channels; C – forward cross-scattering coefficient, which was introduced to address differences in signal attenuation in orthogonal polarization channels and z is the depth coordinate.

Before the linear fit of the logarithmic signal, object surface was found in each dataset and fitting was performed starting at the depth approximately 150 µm from the surface to avoid the influence of the signal reflected from the tissue-air interface.

**Figure 6:**
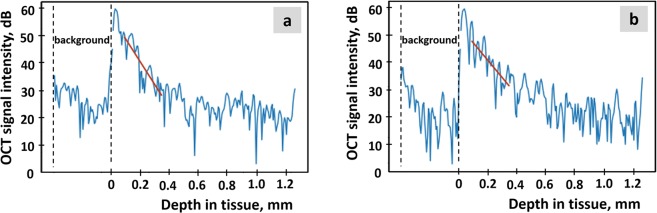
A-scans in co- (**a**) and cross-polarizations (**b**) of a white matter sample on a logarithmic scale (blue lines) and corresponding linear fits (red lines). The attenuation in co-polarization is 4.9 mm^−1^, and the attenuation in cross-polarization is 3.0 mm^−1^. The difference in attenuation coefficients in both polarizations is believed to be caused by forward cross-scattering and is used as a second optical characteristic of the tissue.

A-scans in co-polarization and CP of a white matter sample on a logarithmic scale and corresponding linear fits are shown in Fig. 6. The distributions of the attenuation (ν) and forward cross-scattering (C) coefficients calculated from 3D OCT data volume forms *en-face* color-coded maps.

### Statistical analysis

The median value among all values of optical coefficients calculated for each A-scan of a 3D CP OCT image was used. The results are expressed as the Me [Q1; Q3] where Me is the median of the coefficient and [Q1; Q3] are the 25th and 75th percentile values, respectively. To distinguish tissue types by each coefficient, we used the Mann-Whitney U-test with the hypothesis that there was no difference between the compared groups. An optimal threshold for each coefficient that distinguished tissue types was determined using ROC analysis with maximum specificity and at least 80% sensitivity when possible; otherwise, the optimal threshold was determined based on the requisition of maximal diagnostic accuracy. According to the thresholds for each coefficient, the specificity, sensitivity and diagnostic accuracy were calculated.

## Data Availability

The datasets generated and/or analyzed during the current study are available from the corresponding author on reasonable request.
